# Proteomic Profiling Identifies MARCO in Extracellular Vesicles, as a Potential Biomarker for Leishmaniasis in HIV Co-Infection

**DOI:** 10.3390/ijms26125691

**Published:** 2025-06-13

**Authors:** Inês Costa, Ana Isabel Pinto, Sofia Esteves, Cátia Caldas, Hugo Osório, Nuno Santarém, Carmen Fernandez-Becerra, Anabela Cordeiro-da-Silva

**Affiliations:** 1 Instituto de Investigação e Inovação em Saúde, Universidade do Porto, 4200-135 Porto, Portugal; ines.mcosta21@gmail.com (I.C.); anaisabelpinto@ibmc.up.pt (A.I.P.); cordeiro@i3s.up.pt (A.C.-d.-S.); 2 Laboratório de Microbiologia, Departamento de Ciências Biológicas, Faculdade de Farmácia, Universidade do Porto, 4200-135 Porto, Portugal; 3 Departamento de Doenças Infeciosas, Centro Hospitalar e Universitário de São João, Faculdade de Medicina, Universidade do Porto, 4200-135 Porto, Portugal; 4 Ipatimup–Instituto de Patologia e Imunologia Molecular da Universidade do Porto, 4200-135 Porto, Portugal; 5 Departamento de Patologia, Faculdade de Medicina, Universidade do Porto, 4200-319 Porto, Portugal; 6 ISGlobal, Barcelona Institute for Global Health, Facultat de Medicina i Ciències de la Salut, Universitat de Barcelona (UB), 08007 Barcelona, Spain; 7 IGTP Institut d’Investigació Germans Trias i Pujol, 08916 Badalona, Spain; 8 CIBERINFEC, ISCIII-CIBER de Enfermedades Infecciosas, Instituto de Salud Carlos III, 28029 Madrid, Spain

**Keywords:** *Leishmania*, HIV co-infection, Extracellular Vesicles, Biomarkers, MARCO

## Abstract

*Leishmania* is an intracellular protozoan parasite that causes leishmaniasis, a disease prevalent in 97 countries. Co-infection with HIV increases susceptibility to visceral leishmaniasis (VL), accelerating HIV’s progression to AIDS. Managing VL in HIV-infected individuals is challenging due to atypical presentations and limited therapeutic responses, highlighting the need to develop new disease management strategies. Extracellular vesicles (EVs) hold great promise for this goal as they can be used for a higher understanding of biological processes and biomarker discovery. In this context, a proteomic analysis was carried out from plasma-EVs of an HIV/VL patient over two years and compared to HIV and healthy controls. The analysis confirmed classical EV markers but showed limited detection of *Leishmania* proteins. However, variations in human protein abundance related to relevant immunological processes were observed. Notably, the macrophage receptor with a collagenous structure (MARCO) was consistently detected only in the patient and not in the control groups. Significantly, the relevance of MARCO as a possible VL biomarker was confirmed using a validation cohort with five VL patients and its detection by Western Blot was possible. Although MARCO warrants further investigation as a VL related biomarker, the study of EVs confirmed their promise of being a privileged window into this disease. Future studies are needed to broaden data on EVs in infections to improve clinical management.

## 1. Introduction

Leishmaniasis is the generic name of a group of neglected diseases caused by protozoan parasites from *Leishmania* sp., transmitted to humans by the bite of infected female sandflies. The three most significant forms of leishmaniasis are cutaneous, mucocutaneous, and visceral [1]. The latter, visceral leishmaniasis (VL), is the most severe form, characterized by fever episodes, weight loss, anaemia, and hepatosplenomegaly [2]. VL is highly endemic in the Indian subcontinent and in East Africa, where an estimated 200,000–400,000 new cases occur each year; it is also prevalent in the Mediterranean basin (Portugal, Spain, Greece, and Italy) and South America [1]. *Leishmania*-HIV co-infection has been an emergent problem in the last twenty years and has been reported in 35 endemic countries [3]. HIV-infected people are especially vulnerable to VL, and *Leishmania* infection accelerates HIV replication and advancement to AIDS [3]. Up to 70% of VL cases in southern Europe are associated with HIV infection. In the absence of human vaccines, effective disease management depends on infection detection and treatment. The available therapeutic options are far from optimal, with problems related to their toxicity, high costs, lack of efficacy, and emerging drug resistance. In the context of HIV/VL co-infection, disease presentation is often atypical, and disease management is particularly challenging, with treatment failure and relapses being a common scenario. A better understanding of the disease is essential to develop improved tools for disease management, like new drug or vaccine targets and new biomarkers for disease detection or treatment outcomes. In this context, extracellular vesicles (EVs) are still an untapped resource with great potential. EVs are small lipidic vesicles of cellular origin, present in all biological fluids [4,5,6,7,8]. Several studies have shown that EVs contain proteins and nucleic acids that act as intercellular communicators, with possible relevant roles in many disorders, including infectious diseases and cancer [8]. In the last decade, research on the biology, function, and potential applications of EVs has grown exponentially, expanding to all domains of life. A significant part of the work conducted in this area has shown that perhaps the most important biomedical utility of EVs is their potential as biomarkers for disease management [9]. Compared to other conventional biomarkers detected in biological samples, EVs provide the promise of specificity and sensitivity comparable, or even higher, when compared to their soluble counterparts due to their excellent stability. The release of EVs as part of the *Leishmania* secretion machinery is well-established [10,11]. Furthermore, the protein content of EVs isolated from *Leishmania*-infected macrophages showed modulation upon infection, implying the presence of a potential biological signature linked to infection [8,12,13,14].

The identification of *Leishmania* proteins in EVs recovered in the context of these in vitro infections was also possible [13,14]. Recently, proteomic analysis of plasma-derived EVs from dogs with Canine Leishmaniosis demonstrated that their protein profile was distinct compared to dogs without evidence of infection [15]. The available information in the context of *Leishmania* infection in conjugation with proteomic data on plasma-derived EVs with other related pathogens [16,17,18,19,20] suggests that studying the EVs associated with clinical infections might have a significant biological payoff. Overall, the available data suggest that circulating EVs can contribute to a better understanding of VL infection and could also lead to the discovery of disease-related biomarkers. Thus, the main objective of this study was to find *Leishmania* infection related proteins in plasma EVs in the context of active VL. With this purpose, EVs were isolated in five distinct time points over two years from an HIV-infected patient who was diagnosed with VL in 2013 and had remained infected since then [21]. We then compared these EVs with plasma EVs recovered from control groups without serological and clinical evidence of VL and with and without HIV infection and benchmarked against a validation cohort of five VL patients. With this proteomic comparative analysis, we aimed to identify *Leishmania* and human proteins specifically associated with the VL patient and absent in the control groups as possible biomarkers and to provide exploratory insights into the ongoing immune response. Any possible biomarkers were confirmed in a validation cohort of five VL patients.

Ultimately, these efforts crystalized in the identification of EVs protein profile associated to the patient and more significantly permitted the identification of a possible VL specific biomarker, the protein MARCO (macrophage receptor with a collagenous structure).

## 2. Results

### 2.1. Enzyme-Linked Immunosorbent Assay (ELISA) Results for Antigens SPLA, rK28 and SECA

The parasitological state of the samples from the patient was described elsewhere [21]. The samples used for age- and sex-matched control cohorts (Table S1) did not present seroreactivity suggestive of VL (Figure S1). The serological responses to *Leishmania* antigens relevant for diagnosis were evaluated by Enzyme-Linked Immunosorbent Assay (ELISA) using SPLA, rK28, and the non-related antigen SECA. The seropositivity cut-offs for *Leishmania* were determined in-house and were: SPLA ≥ 0.096; rK28 ≥ 0.098 (Table S2). All samples from the HIV^+^VL^+^ group and validation cohort (Table S3) were above the cut-offs calculated as being seropositive for all the used *Leishmania*-specific antigens (Figure S1).

### 2.2. Purification and Characterization of Plasma-Derived EVs

Human plasma samples from the five different time points of the patient HIV^+^VL^+^ (Table 1) and the HIV^−^VL^−^ and HIV^+^ control groups were processed by size exclusion chromatography (SEC) using one mL sepharose columns.

Fractions of interest were selected based on their characterization by flow cytometry bead-based assay (BBA), using a combination of three of the four makers (CD9, CD81, CD71, and CD5L) and by protein quantification (Figure S2). The fractions with the highest median fluorescent intensity (MFI) in the BBA, enriched in the EV markers, were pooled.

**Figure 1:**
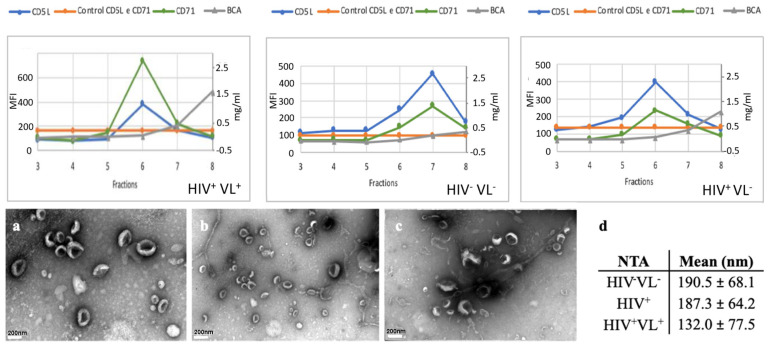
Characterization of EVs. Upper panel: Representative fraction characterization using flow cytometry bead-based assay. SEC fractions 3 through 8 from the patient time points were analyzed using a bead-based flow cytometry for the presence of EV-associated markers CD5L (light blue line). The presence of the antibodies is depicted by absolute MFI plotted on the left y-axis. In the right y-axis is plotted protein concentration for each fraction (BCA, grey line). As negative controls, pooled fractions (5 and 6) beads incubated with secondary antibodies only were used (orange line in the upper graph and light pink in the lower graphs). Lower panel: Representative negative staining of plasma EVs by transmission electron microscopy images. (**a**) HIV^−^VL^−^ sample 1; (**b**) HIV^+^ sample 1; and (**c**) HIV^+^VL^+^ (time point 1). The white bar on the left lower side of each of the three EM pictures represents a 200 nm scale. (**d**) NTA Plasma EVs size evaluation. Mean size in nm and standard deviation determined by NTA for HIV^−^VL^−^, HIV^+^ control groups, and the 5 time points from the patient.

The selected pooled fractions were further characterized by TEM for confirmation of EV presence, by NTA for size determination, and mass spectrometry for identification of *Homo sapiens sapiens* and *Leishmania infantum* proteins. No differences in the morphology of plasma EVs were observed by TEM among the different groups of EV samples analyzed (Figure 1a–c). The average sizes determined by NTA were between 132.0 and 190.0 nm (Figure 1d).

### 2.3. Leishmania Proteins Identification in Patient HIV^+^/VL^+^

The detection of *Leishmania* proteins associated with plasma-derived EVs was a primary objective. For this, the plasma EVs recovered at the different time points of the HIV^+^VL^+^ patient were analysed by mass spectrometry to identify peptides associated with *Leishmania* proteins (Table 2).

These were associated with 1 UP identification, and no protein was common between different time points of the patient. Except for the putative RNA helicase, all the peptides reported are 100% specific for *Leishmania* upon BLAST analysis. No *Leishmania* proteins were detected in the HIV^−^VL^−^ cohort. Interestingly, in the validation cohort, three single peptide identifications associated with *Leishmania*, with 1 UP and 1 PSM, were found. In a subsequent analysis from a pooled sample containing all HIV^+^/VL^+^ five time points, using 100 μL from each time point, eight other *Leishmania* proteins were identified (Table 2). Two of these proteins identified in the pool were also detected in Sample 3, HIV^+^VL^+^, from the validation group (Table 2 and Table S4). When these identifications were compared to the *Leishmania* protein data set obtained from plasma EVs recovered from Canine Leishmaniosis dogs, no similar proteins were identified [15].

### 2.4. Human Proteins Identification in Patient HIV^+^VL^+^

**Figure 2:**
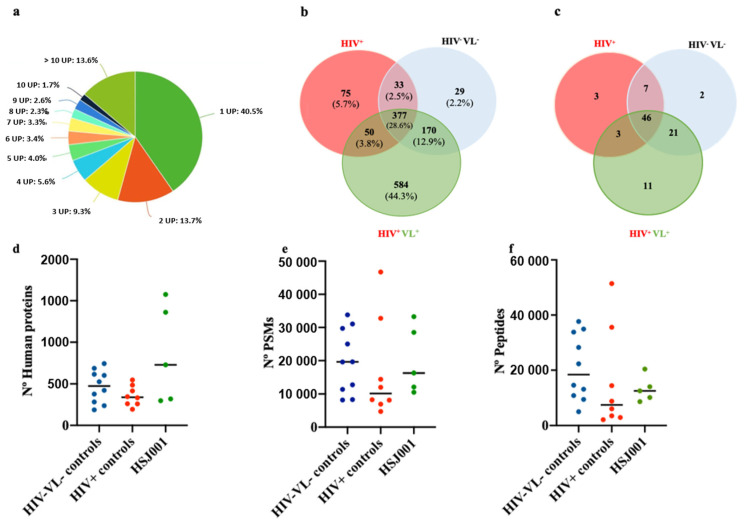
Quantitative proteomic analysis. (**a**) Percentage of unique peptide number associated with each protein identification for plasma EVs recovered in the study. (**b**) Comparative protein identifications Venn diagram illustrating the overlap in number and percentage of human proteins identified in the patient time points (HIV^+^VL^+^—green circle), and in both control groups, the HIV^+^—red circle and HIV^−^VL^−^—blue circle. (**c**): Venn diagram illustrating the overlap in protein identifications associated with groups 1a, 1b; 2a, 2b, and 3 of the MISEV2018 guidelines, in the patient time points (HIV^+^VL^+^), and in both control groups, the HIV^+^ and HIV^−^VL^−^. Number of human proteins (**d**), peptide-spectrum match—PSMs (**e**), and peptides (**f**) identified. Each dot represents an individual plasma EV sample from the control groups or the patient time points (HIV^+^/VL^+^). The horizontal bars represent the average of the group. Statistically significant differences between the groups were not observed using the Kruskal–Wallis test.

For the proteomic analysis, only proteins with ≥2UP were considered. Thus, only 59.5% of the protein identifications were used in subsequent analysis (Figure 2a).

Overall, 1318 human proteins were identified in the individual samples, considering the threshold of at least two unique peptides (Figure 2b). Considering the protein identifications, from the control groups and the patient, 28.6% of the protein identifications were consistently detected in all samples using the merged data constituting the core proteome (Figure 2b). Among these three study groups, the patient had the most unique protein profile, with 584 proteins (44.3% of the total protein identifications) absent in control groups (Figure 2b).

According to the MISEV2018 guidelines [22], all EVs preparations have more than two identifications for categories 1 and 2 (Figure 2c and Figure S3). Still, the fractions are not pure, as they contain proteins from categories 3, 4, and 5 (Figures S3 and S4). Regarding the recommended EVs markers, among the transmembrane or GPI-anchored proteins associated with the plasma membrane and/or endosomes, the integrin alpha (ITGA) and beta (ITGB) were the most consistently identified in the individual samples (Figure S3). Integrin alpha-IIb ITGA2B was the most consistently detected. The monocyte differentiation antigen CD14 was detected in only three out of five time points of the patient HIV^+^VL^+^. Among the cytosolic proteins recovered in EVs, the Heat shock 70 kDa protein 1B HSPA1B was the most consistently detected. Several non-EV co-isolated molecules were also detected, like the Apolipoprotein A-I (APOA1) detected in all samples, except in one (Figure S3). Among the markers used in the bead-based assay, CD9 and CD5L were the most consistently detected in the proteomic evaluation. CD5L was detected in 21/23 main study samples (Figure S4).

In the patient, an average of 727 proteins were identified among the five time points. For the controls, an average of 474 and 339 human proteins were detected in the HIV^−^VL^−^ and HIV^+^ control groups, respectively (Figure 2d). HIV proteins were not detected in any of the groups. No significant differences existed in terms of overall protein identifications, peptide numbers, or PSMs between the time points of the patient and the control groups (Figure 2d–f).

### 2.5. Quantitative Analysis of Human Proteins

**Figure 3:**
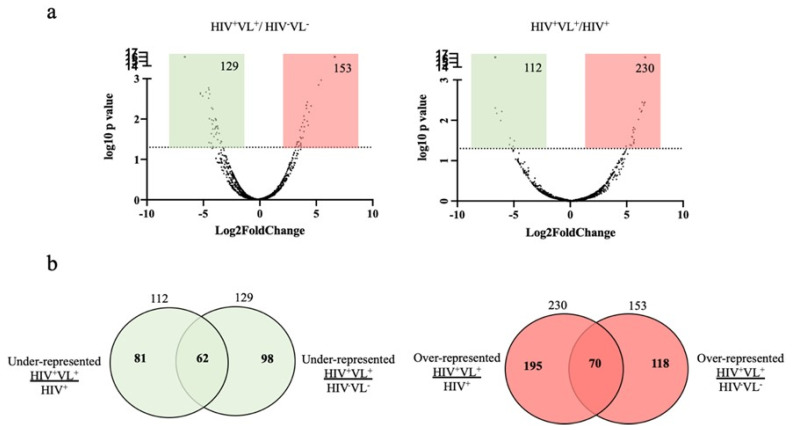
Quantitative comparison of detection in the patient and control groups. (**a**) Relative abundance of protein identifications in the different groups. Volcano plot representation of the abundance ratio of proteins present in ratios HIV^+^VL^+^/HIV^−^VL^−^ and HIV^+^VL^+^/HIV^+^. The differentially abundant proteins (*p* < 0.05) are represented in coloured boxes. In red—protein identifications significantly more abundant in the numerator of each ratio represented; in green—protein identifications significantly less abundant in the numerator of each ratio represented. (**b**) Venn diagram illustrating the number and percentage of human proteins detected that are significantly underrepresented (green) or overrepresented (red) in the patient when compared to HIV^+^ or HIV^−^ VL^−^ control groups.

A quantitative analysis was also conducted to compare the protein abundances among the patient samples at different time points (HIV^+^/VL^+^) with those of the two control groups. Upon considering the abundance ratios associated with the protein identifications among the three groups, it became evident that several proteins were significantly overrepresented or underrepresented in each comparison (Figure 3a). In fact, 153 and 230 proteins were significantly overrepresented in the patient time points when compared to HIV^−^VL^−^ and HIV^+^ controls, respectively (Figure 3a). Moreover, when we compared the proteins from the patient that were underrepresented or overrepresented to the control groups, 62 protein identifications were identified as being significantly underrepresented when compared to both control groups (Figure 3b, Table S5). On the other hand, 70 were significantly overrepresented (Figure 3b, Table S6).

**Figure 4:**
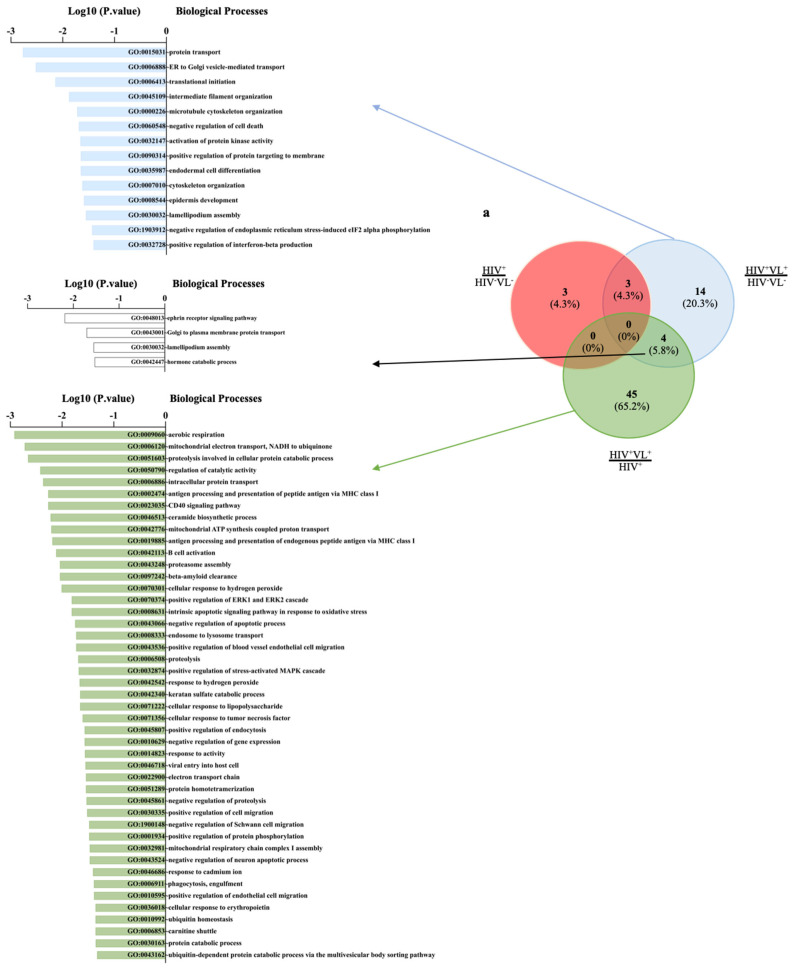

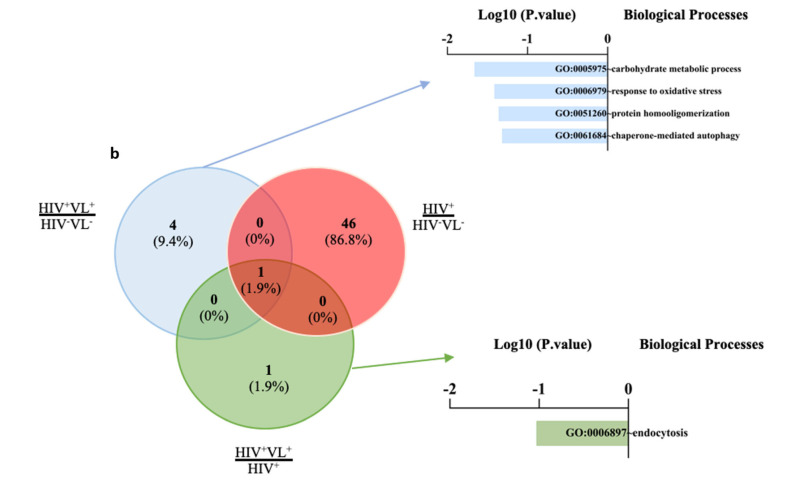
Distribution of biological processes identified by Gene Ontology (GO). GO enrichment analysis associated with proteins overrepresented or underrepresented in the patient (HIV^+^/VL^+^) when compared to HIV^+^ or HIV^−^VL^−^ controls. In (**a**), the Venn diagram and associated graphs, listing GO identifications and *p*-values, illustrate the distribution of biological processes identified by GO analysis using proteins overrepresented in the patient compared to the HIV^−^VL^−^ controls (blue circle) or to the VL^−^ control (green circle). The red circle represents the same analysis using proteins overrepresented in the HIV^+^ control group compared to the HIV^−^VL^−^ controls. In (**b**), the Venn diagram and associated graphs, listing GO identifications and *p*-values, illustrate the distribution of biological processes identified by GO analysis using proteins underrepresented in the patient compared to the HIV^−^VL^−^ controls (blue circle) or to the VL^−^ control (green circle). The red circle represents the same analysis using proteins underrepresented in the HIV^+^ control group compared to the HIV^−^VL^−^ controls. Only GO terms with *p*-value < 0.05 are considered. List of Biological Processes associated with (**a**,**b**) include the Log10 of the *p*-value.

Considering the distinct pattern of protein abundance in plasma EVs, when comparing the patient and the control groups (Figure 3a), a gene ontology enrichment analysis was performed. This approach used the distinct subsets of proteins underrepresented or overrepresented when comparing the patient with the controls (Figure 3b). Considering the 230 proteins that were significantly more abundant in the patient in comparison to the HIV^+^ control group, 49 biological processes were significantly overrepresented using DAVID (Figure 4a, Table S7).

Comparing the biological processes associated with proteins overrepresented in the patient compared with the control groups, only four biological processes were retained (Figure 4a). Several biological processes of immunological interest were associated with the proteins of enriched abundance in the patient in comparison with the HIV group (Figure 4a). Among these, we highlight the following: GO:0009060~aerobic respiration (*p*-value 0.001); GO:0042113~B cell activation (*p*-value 0.008); GO:0070301~cellular response to hydrogen peroxide (*p*-value 0.010); GO:0023035~CD40 signalling pathway (*p*-value 0.005); GO:0071222~cellular response to lipopolysaccharide (*p*-value 0.022); GO:0006911~phagocytosis, engulfment (*p*-value 0.041); GO:0002474~antigen processing and presentation of peptide antigen via MHC class I (*p*-value 0.005) and GO:0019885~antigen processing and presentation of endogenous peptide antigen via MHC class I (*p*-value 0.007). When considering the same GO analysis for the proteins significantly underrepresented in the patient compared to control groups, only one biological process was identified in HIV^+^ and four for the HIV^−^VL^−^ (Figure 4b). The list of biological processes is represented in Table S7.

### 2.6. Human Derived Biomarkers of VL

**Figure 5:**
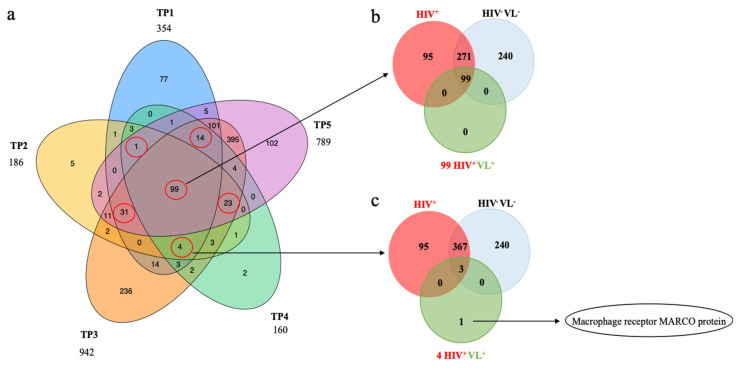
(**a**) Identifications of proteins present in the patient and absent in the control groups. Comparison of protein identifications from different time points of the patient HIV^+^VL^+^. Venn diagram illustrating the number of human proteins in each different time point of the patient with more than one unique peptide and a maximum false discovery rate (FDR) of 1%. The red circles highlight the number of common protein identifications in the Venn diagram intersections with 4 or 5 patient time points. These were subjected to comparison with the HIV^+^ and HIV^+^/VL^−^ control groups as depicted in (**b**,**c**). (**b**) The upper Venn diagram represents the identification overlap between all 99 proteins in common in the different time points of the patient HIV^+^VL^+^ (in green) and the control groups HIV^+^ (in red) and HIV^−^VL^−^ (in blue). (**c**) The lower Venn diagram represents the identification overlap between the selected four proteins in common to four different time points of the patient (in green) and the control groups HIV^+^ (in red); HIV^−^VL^−^ (in blue). The protein unique to the patient was identified as the Macrophage receptor MARCO protein.

Ideally, proteins that could serve as biomarkers should be detected in the patient and not in the control groups. To evaluate if any particular human protein identified in plasma-derived EVs was associated with the patient HIV^+^VL^+^ and not the control groups, we compared the individual identifications of the patient time points (Figure 5a) to identify common proteins.

**Figure 6:**
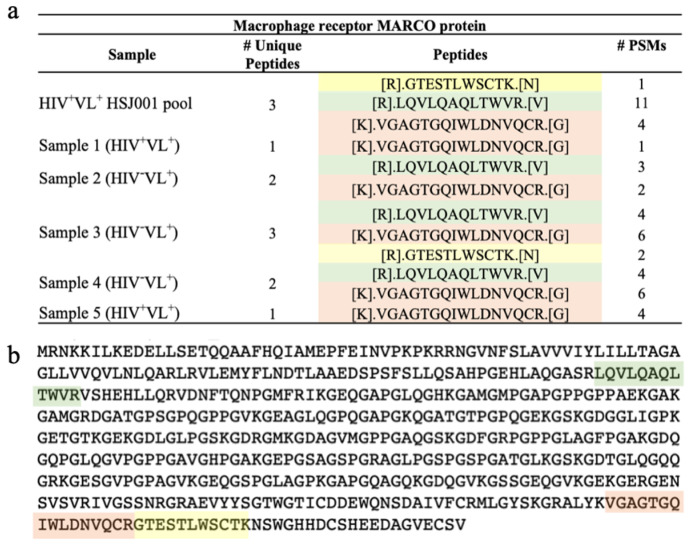
Human protein macrophage receptor MARCO identification in validation cohort. Depicted in the upper panel (**a**) is a table representing the unique peptides (UP), peptide sequence, and protein spectral matches (PSMs) unique peptides associated with the protein identification in the samples from the pool of the patient and validation cohort. Each one of the three UP identified is associated to a distinct colour. The lower panel (**b**) depicts the macrophage receptor MARCO protein sequence and peptides identified associated with the plasma EV samples colour-coded to match the peptide sequences in panel (**a**). MARCO characteristics: Length: 520; Mass (Da): 52,658. Isoelectric point: 8.95.

Ninety-nine proteins were found in common with all different time points of the patient (Figure 5a). These 99 proteins were also present in the core proteome of 377 defined for all the plasma EVs (Figure 2b and Figure 5). Among the proteins detected in four out of five time points, only one was not detected in the control groups (and also not detected in time-point 5) (Figure 5c). This protein was the macrophage receptor with collagenous structure (MARCO) (Figure 6).

To confirm MARCO as a VL-specific biomarker, a pool of plasma from the five time points and also from healthy donors was used for a new plasma EVs recovery. The proteomic analysis confirmed the detection of this macrophage receptor with three UP and 16 PSMs in the pool and not in the control pools. To further confirm MARCO as a VL relevant identification, we used our validation group with five other VL patients (Table S2). MARCO was also detected in our validation cohort in three samples with two or more UP and only 1 UP in the other two (Figure 6). To evaluate if MARCO was a common identification in the context of other infectious diseases, we searched for it on proteomic data sets from patients with malaria and Chagas disease that were recovered using a similar approach [17,23] and also in the available Vesiclepedia dataset [24]. No peptide identifications consistent with MARCO were present in malaria and Chagas patients and only one study reported the identification of MARCO in Vesiclepedia [25]. Considering that Leishmaniasis in Portugal is a zoonotic disease, dogs being the natural reservoir, it was also evaluated if MARCO was identified in plasma EVs from dogs with Canine Leishmaniosis from a previous proteomic study using the same EVs recovery approach [15]. A unique peptide consistent with MARCO was detected in four infected dogs (Table S8). It was not detected in non-infected dogs.

**Figure 7:**
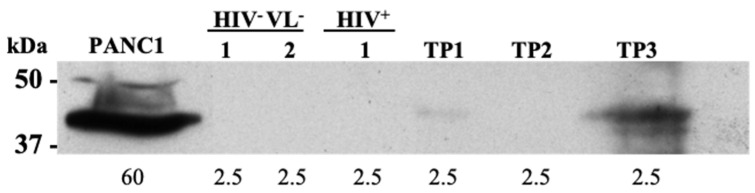
Detection of MARCO by Western blot in plasma-derived EVs. MARCO antibody was used to detect MARCO in the plasma EVs from three time points from the patient HIV+VL+, time-points TP1, TP2, and TP3, and also in three controls (2 HIV-VL- and 1 HIV+). PANC1 cell extract was used as a positive control. The protein amount in μg loaded on the gel is shown under each lane.

As a proof of concept for MARCO detection in plasma EVs, a commercially available anti-MARCO was used to perform WB on the plasma-derived EVs. MARCO was detected in time points one and three of the patient (Figure 7). No detection of MARCO was observed in the selected samples representing the control groups HIV^−^VL^−^ and HIV^+^ (Figure 7). MARCO was also detected in time-point two after a new plasma EVs purification and concentration of EV fractions (Figure S5).

## 3. Discussion

Understanding the ongoing immune response and the interaction between the host and pathogen during co-infections of *Leishmania* and HIV is crucial for developing new strategies for disease management in this highly vulnerable population. The proteomic analysis of circulating plasma EVs can not only provide insights into ongoing physiological processes but also lead to the discovery of disease-specific biomarkers that could be useful for managing these infections. Plasma from a recently described case report of a Portuguese patient with a long-lasting VL-HIV infection [21] was utilized to explore, for the first time, the potential of plasma-derived EVs in managing HIV-VL co-infections. Identifying proteins associated with *L. infantum* would be highly valuable, not only for enhancing our understanding of the infection but also as potential biomarkers for exploitation. However, only one unique peptide was detected for each protein identified, and most proteins were detected in only one sample, which limits conclusive protein identification. This limitation was also noted when using the same methodology to isolate plasma-derived EVs for identifying *T. cruzi* biomarkers in a heart transplant patient with chronic Chagas disease, as well as in the context of canine leishmaniasis [15,17]. One possible explanation for these findings is that the parasite-derived peptides required for identification are masked by much more abundant host proteins. Furthermore, since *Leishmania* is an intracellular pathogen in the mammalian host, there will be less free parasitic material released directly into the plasma, making it difficult to identify parasite-associated proteins in circulating plasma EVs. Moreover, the fact that *Leishmania* resides within the phagolysosomes of specific cell types might further hinder the access of parasite derived material to the plasma. Notwithstanding, it is conceivable that *Leishmania* proteins could access the endosomal machinery through fusion events that allow these proteins to enter the macrophage cytosol or via direct externalization of EVs into the cytosol [26,27]. These EVs might then be released inside host EVs in a process akin to transcytosis, which has been previously documented for other pathogens [28,29]. Therefore, it is plausible that a similar occurrence could happen with pathogen-specific EVs. A reassessment of the composition of circulating plasma EVs in *Leishmania* infections adapting immunoaffinity capture techniques that utilize specific markers enriched in EVs produced by *Leishmania* infected cells might improve the capacity to detect parasite proteins.

The recovery of plasma EVs in the context of HIV-VL co-infection enabled the detection of over 1318 human proteins. Notably, no significant differences in overall total protein identifications, peptide numbers, and PSMs were observed comparing the patient with control groups. This provided more confidence to the qualitative and quantitative analyses performed. If significant changes had been present, they could have introduced a strong bias in protein identification and quantification. Interestingly, a similar recovery method produced comparable results when examining plasma EVs from dogs, regardless of whether they showed evidence of *Leishmania* infection. [15]. Our findings contrast with other proteomic analyses of plasma-derived EVs in malaria, which reported a higher total number of proteins in EVs from healthy donors compared to those from infected individuals [23]. Furthermore, the total number of recovered EVs using the same technique for these two distinct hosts suggests that the overall amount of EVs may not be a significant factor in disease manifestation associated with *L. infantum* infection [23]. This is in contrast to other infectious diseases, which used different EV recovery methods, which reported variations in the number of EVs present in circulation [30].

The plasma EVs fractions recovered contain structures similar to EVs and are enriched in EVs-associated proteins. Considering the MISEV2018 recommended EVs markers, specifically the transmembrane or GPI-anchored proteins associated with the plasma membrane and/or endosomes, integrin alpha (ITGA) and integrin beta (ITGB) were the most consistently identified across individual samples. Others, like Integrin beta-3 ITGB3, were more frequently detected in a specific group, in this case, the HIV^−^VL^−^ cohort. This suggests that while the overall EV number may not vary significantly, the composition of the plasma EV population may differ upon infection. Similar findings were reported in plasma samples from dogs with canine leishmaniosis, where CD82 was enriched in the diseased cohort [15]. Both integrin alpha and beta were consistently detected in individual canine samples. Additionally, the heat shock protein family A member 8 (HSPA8) was detected in most samples [15]. Among the cytosolic proteins recovered from plasma EVs, the heat shock 70 kDa protein 1B (HSPA1B) was the most frequently identified. Several non-EV co-isolated structures were also found, with apolipoprotein A-I (APOA1) detected in all but one sample. Concerning the markers CD5L and CD9, these were identified in most samples using our flow cytometry bead-based assay. Although CD5L is described as a secreted protein that co-isolates with EVs, it is also reported as an exosomal marker of plasma-derived EVs in human samples [31]. This marker was found in high abundance across most samples, similar to the enrichment of CD5L in canine leishmaniasis samples [15]. Interestingly, the monocyte differentiation antigen CD14 was only detected in the study patient, being detected in three of the five time points. Although this observation can be patient specific, CD14 is associated with circulating monocytes and macrophages, both of which are traditional hosts for *Leishmania* infection. Other studies have reported elevated levels of soluble CD14 in VL patients, suggesting its possible involvement in the immune response to VL [32]. Moreover, soluble CD14 levels correlated with mortality during the chronic phase of HIV infection, with baseline CD14 levels associated with a more rapid decline in CD4 cells and a higher risk of death from coronary heart disease [33]. Given that these cells are natural hosts for *Leishmania* and that CD14 can be found in exosomes, investigating CD14 for a potential positive selection approach is warranted. One overarching goal of studying plasma-derived EVs from a VL-co-infected HIV clinical case was to assess the biological impact of this co-infection through Gene Ontology (GO) enrichment analysis. Once again, these observations must take into consideration that they could represent the specific condition of the patient and not *Leishmania* infection specific effects. Several immunologically significant biological processes were enriched in this patient compared to the HIV^+^ control group.

Notably, “antigen processing and presentation of peptide antigens via MHC class I” (GO:0002474) and “antigen processing and presentation of endogenous peptide antigens via MHC class I” (GO:0019885) were enriched in the patient’s plasma EVs. This process was also reported as a GO enrichment in plasma EVs from canine leishmaniasis [15]. The delivery of proteins associated with MHC class I presentation through exosomes has been observed in other pathogens, such as *M. tuberculosis* [34]. In fact, antigen presentation through exosomes from infected cells may serve as an alternative mechanism for cross-presentation, thereby inducing an acquired immune response [34]. The “cellular response to lipopolysaccharide” (GO:0071222) was also increased in the patient. The role of Toll-like receptor 4 (TLR4) in controlling *Leishmania* infection is well-documented. TLR4-competent mice exhibit enhanced nitric oxide production, which limits parasite growth, while TLR4-deficient mice show increased arginase activity, promoting parasite replication [35]. Associated with the cellular response to lipopolysaccharide, the protein arginase-1 (Arg1) was identified, comprising four unique peptides and four peptide spectrum matches. The expression of this protein has been linked to disease susceptibility and chronicity in *Leishmania* infections [36]. In vivo studies have shown that bone marrow-derived macrophages from susceptible BALB/c mice exhibit higher arginase activity and parasite growth upon Th2 cytokine stimulation compared to resistant C57BL/6 mice, establishing a positive correlation between *Leishmania* growth and Arg1 activity [37]. Furthermore, arginase activity has been associated with *L. major* growth and pathology in younger mice [37], with increased expression correlating with susceptibility and higher parasite loads in susceptible mice, while having reduced expression associated with lesion resolution and healing in resistant mice [38,39]. Additionally, the biological process “negative regulation of apoptotic process” was associated with 13 protein identifications in the patient-derived EVs. *Leishmania* employs various strategies to evade apoptosis and persist within host cells, including inhibiting apoptosis to downregulate host cell defence mechanisms and perpetuate infection [40]. These GO observations underscore the biological relevance of studying plasma EVs, highlighting the significance of their associated proteins in understanding the immune response to infections.

Although definitive identifications of parasite proteins were not possible, a host protein known as the Macrophage receptor with collagenous structure (MARCO) was consistently detected in the patient samples but was absent in the HIV controls. To further validate the presence of MARCO, a pooled analysis of all time points for the patient was conducted. Three unique peptides were identified, with 1, 4, and 11 PSMs corresponding to each peptide, respectively. A similar analysis of healthy samples revealed that MARCO was not detected in those samples. The relevance of MARCO protein identification was confirmed using a validation cohort of five other patients with VL. In fact, MARCO peptides were identified in the five patients. This observation was essential to exclude patient specific identification of MARCO. Additionally, it was detected in plasma EVs isolated from dogs infected with *Leishmania*, while it was not found in the negative controls [15]. Western blotting was used not only to confirm the presence of MARCO in the patient samples, but more significantly to demonstrate that MARCO can be detected using specific antibodies in EVs preparations opening the door to future clinical applications. Although the studies were limited by the availability of plasma EVs from the patient for repeat analyses, the consistent detection of MARCO suggests that it can be reliably identified plasma EVs samples. Notably, at time point five for the patient, where MARCO could not be identified by MS, there was a significant ongoing immune response indicated by plasmatic cytokines, alongside a reduced *Leishmania*-specific cellular response [21]. This may suggest that an ongoing immune response to another pathogen was masking the detection of MARCO. The Monocyte Chemotactic Protein 1 (MCP-1) was proposed as a biomarker for the cellular immune response to *Leishmania* [41]. MCP-1 was produced upon whole blood stimulation in time points 1 through 4 but was absent at time point 5 [21]. Interestingly, it was also at this time point that MARCO was not detected by MS. The similar profiles of MARCO and MCP-1 suggest that MARCO may also serve as a biomarker for the *Leishmania*-specific immune response.

A major concern is that, although MARCO was not detected in the HIV^+^ control cohort, it could still be a marker associated with non-viral infections. Nevertheless, MARCO was also not found in plasma EVs from patients with Chagas disease or malaria using the same EV recovery methods [17,20,23]. Interestingly, according to the Vesiclepedia database, MARCO has only been reported once in relation to a tuberculosis patient [25]. This information is particularly noteworthy because *M. tuberculosis* is an intracellular pathogen that specifically infects macrophages. This raises the possibility that MARCO might be associated with pathogens that target macrophages. MARCO is a class A scavenger receptor located on specific subsets of macrophages [42]. Scavenger receptors are pattern-recognition receptors present in immune cells [43]. It has been reported that MARCO may play a role in the uptake of exosomes through dynamin-dependent endocytosis and micropinocytosis [44]. Additionally, MARCO is capable of binding to and phagocytosing pathogen-associated molecular patterns [45].

As a component of the innate immune system, MARCO plays a role in pathogen clearance and inflammatory responses [46]. MARCO is one of the most differentially expressed genes found in yolk-sac-derived Kupffer cells, showing a 15-fold increase in mRNA abundance compared to bone marrow-derived macrophages [47]. Interestingly, most tissue-resident macrophages stem from the yolk sac and bypass the classical monocyte intermediates, retaining the ability to self-renew throughout the lifespan [48,49]. Additionally, MARCO expression is upregulated by various Th1-polarizing factors while it is downregulated by Th2-polarizing factors [50]. Thus, MARCO protein in plasma EVs might result from ongoing exposure to *Leishmania*-associated pathogen-associated molecular patterns (PAMPs) in the liver, where yolk-sac-derived macrophages, which express higher levels of MARCO, are prevalent and heavily infected [51].

Interestingly, MARCO has been studied in *Leishmania* infection. Importantly an anti-MARCO monoclonal antibody reduced *L. major* infection of macrophages by 30–40% in vitro. They suggest that MARCO has a role in macrophage infection by *L. major* in vitro as well as in vivo, as lymph nodes of anti-MARCO-treated mice displayed a reduced presence of immunolabelled parasite and parasite antigens, as well as a reduced inflammatory response [52]. Interestingly, CBA/J mice are resistant to *Leishmania major* but susceptible to *L. amazonensis*. CBA/J macrophages can control *L. major* infection but not *L. amazonensis* infection in vitro. Studies show that MARCO expression in CBA/J macrophages increases in response to both in vitro and in vivo *L. major* infections, but not to *L. amazonensis* [52]. This suggests species specific modulation of MARCO, something that must be addressed in subsequent studies. Research on other pathogens like *Neisseria meningitidis* also identified MARCO as a marker for innate macrophage activation [53]. Both mouse and human MARCO can bind to *N. meningitidis* independently of lipopolysaccharides (LPS), suggesting that TLR-dependent induction of MARCO through innate immune stimulation enhances the recognition and uptake of pathogenic organisms, thereby contributing to host defence against infection [53]. MARCO expression on macrophages has also been linked to tumour development. In cases of hepatocellular carcinoma, MARCO was among the top 30 differentially expressed genes when comparing cancerous tissues to adjacent non-cancerous tissues [54]. Overall, the known biology of MARCO presents characteristics of inducibility that could be explored as potential biomarkers.

Ultimately, the data presented in this study highlight, for the first time, that plasma-derived EVs may serve as valuable predictors of ongoing infection-related physiological processes in VL. Additionally, the changes in EV markers observed during infection could be used to define future Leishmaniasis specific positive selection methods for EVs recovery. This investigation of plasma-derived EVs in the context of HIV/VL co-infection shows promise in advancing our understanding of this disease. Further validation is needed to establish the potential role of MARCO in VL management. Confirming its presence on the surface of EVs would be crucial for proposing MARCO as a potential biomarker for VL patients. Moreover, studying larger groups of VL patients, as well as individuals with pathogens with similar lifestyles such as *M. tuberculosis* infections, is necessary to validate MARCO’s potential as a tool for VL management.

## 4. Materials and Methods

### 4.1. Clinical Description of the Study Patient

The patient was a 45-year-old male who resided in Vila Nova Gaia, a traditionally non-endemic area for leishmaniasis [55]. The detailed clinical description is reported elsewhere [21]. The patient was diagnosed with human immunodeficiency virus (HIV) in March 2000. In 2013, he was diagnosed with Visceral Leishmaniasis (VL). Despite treatment and prophylaxis with anti-leishmanials, the patient remained infected with *Leishmania* until passing away in 2022. We had access to plasma samples from the patient between 2019 and 2021 (Table 1).

### 4.2. Cohort Design: Leishmania^+^HIV^+^ Patient, HIV^+^ Patients and HIV^−^VL^−^ Controls

The study included the groups:

HIV^+^VL^+^: Plasma samples from the patient were collected at five distinct time points with an average interval of six months between 2019 and 2021 (Table 1).

HIV^+^: Control plasma samples from HIV-positive individuals (n = 8) from the Centro Hospitalar Universitário São João (CHUSJ), with an average age of 51 ± 4.6 and sex paired with the study patient (Table S1).

HIV^−^VL^−^: Control plasma samples (n = 10) from the CHUSJ, with an average age of 47 ± 4.5 and sex paired with the study patient (Table S1).

Validation cohort: Plasma from five other VL patients, two of whom are also HIV positive, with different ages and sex from Portugal and Spain were also used to validate the detection of MARCO protein by Mass Spectrometry (MS) (Table S3).

Serum samples for serological evaluation of soluble promastigote *Leishmania* antigens (SPLA) and rK28: For seropositivity cut-off determination of SPLA and rK28 the control groups were VL^+^ (n = 52) and VL^−^ (n = 52). VL^+^ samples were confirmed cases of VL by either parasitological or serological/cellular evidence. All samples were collected in Spain by Instituto de Salud Carlos III (ISCIII). Negative samples were obtained from the Madrid blood bank.

### 4.3. Blood Collection and Plasma Processing

Total blood and plasma were collected from all study participants. Blood samples were centrifuged at 800× *g* for 20 min at 4 °C, and the plasma was collected and stored at −80 °C until further analysis.

### 4.4. Parasites and Cell Culture

*Leishmania infantum* (MHOM/MA/67/ITMAP-263) promastigotes recovered from spleen of infected mice were maintained in standard RPMI 1640 medium supplemented with 10% Fetal Bovine Serum (FBS), 2 mM L-glutamine, 100 U/mL penicillin, 100 mg/mL streptomycin, and 20 mM HEPES buffer (all products from Lonza, Basel, Switzerland) at 26 °C. Weekly passages with a starting inoculum of 1 × 10^6^ parasites/mL were used for no longer than 10 passages [56,57].

### 4.5. Antigens

For SPLA production, five-day-old promastigotes were washed three times with PBS and centrifuged at 3500× *g* for 10 min at 4 °C. The pellet was resuspended in PBS containing 1 mM phenylmethylsulphonyl fluoride (PMSF) protease inhibitor and subjected to 10 freeze-thaw cycles to induce rupture of the parasites. The suspension was centrifuged at 13,000× *g* for 30 min at 4 °C and the supernatant was recovered, quantified by DC (detergent compatible) protein assay (Bio-Rad Laboratories, Hercules, CA, USA), and stored at −80 °C in single aliquots.

The rK28, obtained from Dr. Steven Reed (Infectious Disease Research Institute, Seattle, WA, USA), was resuspended in water, quantified, and stored at −80 °C in single use aliquots.

### 4.6. Enzyme-Linked Immunosorbent Assay (ELISA)

All samples included in the study were evaluated by ELISA for their seroreactivity against *Leishmania* antigens. The *Leishmania*-specific selected antigens were rK28 (kinesin-related protein of *L. infantum* [58] and the soluble promastigote *Leishmania* antigen (SPLA). A non-related antigen, the soluble *Escherichia coli* antigen (SECA), was also evaluated [59]. The coating was conducted overnight at 4 °C with 50 µL of coating buffer (0.05 M carbonate/bicarbonate pH 9.6) containing 5 µg/mL (rK28) or 10 µg/mL (SPLA or SECA) antigens. On the next day, the antigens were discarded and 200 µL of PBS with 10% milk was added to block the plate for 60 min at 37 °C. After washing 4× with PBS-tween 0.05%, 100 µL of the sera diluted 1:400 in PBS-tween 1% milk was added to the wells. A positive control was always added at 1:500 in one well. Incubate at 37 °C for 60 min. After four washes, the secondary antibody (Anti-human IgG (whole molecule) Peroxidase antibody, A8667, Sigma Aldrich, Darmstadt, Germany) at 100 µL per well at a 1:5000 dilution in PBS-tween 1% milk and incubated for 60 min at 37 °C. After four washes, 100 µL of OPD (5 mg tablet in 10 mL of citrate (0.05 M Na_2_HPO_4_; 0.02 M citric acid) + 10 µL H_2_O_2_) was added. After 10 min, the reaction was stopped by adding 50 µL of 3M HCL. Absorbance was read at 492 nm in an automatic reader (Synergy 2, Agilent Bio Tek Instruments, Santa Clara, CA, USA). All samples and antigens were assayed in at least two independent assays.

### 4.7. Roc Curve Determination

Receiver operating characteristic (ROC) curves were generated using sera from VL^+^ and VL^−^ groups. A 95% confidence interval (95% CI) for the area under the curve (AUC) was considered. Cut-off values were inferred through these curves for each antigen (by choosing the best compromise between sensitivity and specificity associated with the ROC curve), and values of sensitivity (Se) and specificity (Sp) were calculated for the samples for each group using Prism 9 for Windows version 9.4.0.

### 4.8. Purification of Plasma-Derived EVs by Size-Exclusion Chromatograph

The purification of plasma EVs from the different patients was performed by size exclusion chromatography (SEC), following established procedures [31]. Briefly, Sepharose CL-2B (Sigma CL2B300 Darmstadt, Germany) was loaded into a 1 mL disposable syringe with an eccentric tip (BD 300013, 3-way valve for syringe BD 394600 Franklin Lakes, NJ, USA) and packed in sterile conditions one day before the isolation to a final volume of 1 mL; the syringe tip was sealed by adding a fragment of sterile nylon stocking. Columns were equilibrated with PBS and were kept at 4 °C until use. The day after, plasma was processed by centrifugation at 2000 g for 10 min at 4 °C. Then, 100 μL of the plasma was loaded, and ten fractions (of 100 μL each) were collected for later analysis or frozen at −80 °C. Five individual columns were used for each sample.

### 4.9. Bead-Based Flow Cytometry Assay

Plasma-derived EVs were characterized by bead-based flow cytometry assay using specific exosomal markers, such as CD5L, CD71, CD81, and CD9, as previously described [23,31,60] with some modifications. Anti-CD5L antibody (Abcam: ab45408, Cambridge, UK) at 1:1000 dilution, anti-CD71 antibody (Abcam: ab84036) at 1:1000 dilution, anti-CD9 (Immunostep 9PU-01MG, Salamanca, Spain) at 1:500 dilution and anti-CD81 Santa Cruz Biotechnology (Sc-23962), Santa Cruz, California at 1:500 dilution were used for 30 min at 4 °C in a round bottom plastic microplate. SEC fractions were coupled to Aldehyde/Sulfate Latex Beads, 4% *w/v* (Invitrogen, Carlsbad, CA, USA), by incubation for 15 min with agitation. Coupled beads were then blocked by incubation overnight with 1 mL of BCB Buffer (PBS-BSA 0.1%) in a rotary shaker. Beads were further centrifuged at 2000× *g* for 10 min, supernatants were removed and pelleted beads were resuspended in BCB buffer. The beads suspension was analyzed to study the presence of classical EV markers being incubated with primary antibodies diluted with BCB buffer for 30 min at 4 °C. After washing with BCB, EV-coated beads were incubated for 1 h at 4 °C with secondary antibodies made in rabbit or mouse (Goat anti-mouse IgG 1:100 Southern Biotech 1032-02, Birmingham, AL, USA; Goat anti-rabbit IgG 1:500 Invitrogen A11008, Carlsbad, CA, USA). Negative controls included SEC fractions predicted to contain a high concentration of EVs incubated only with the respective secondary antibodies. Labelled EV-beads were washed twice with BCB before being finally resuspended in PBS and analysed in an Accuri C6 cytometer (BD Life Sciences, Franklin Lakes, NJ, USA). FlowJo V10 software (Tree Star, Woodburn, OR, USA) was used to compare the mean fluorescence intensity (MFI) of bead populations between EV preparations. The fractions with the highest MFI in the bead-based assay, enriched in the EV markers, were pooled for further analysis.

### 4.10. Plasma EVs Protein Quantification

The protein concentration of the pooled fractions was determined by the BCA protein assay kit (Pierce, Thermo Fisher Scientific, Waltham, MA USA) following the manufacturer’s instructions. To begin, 10 μL of each standard or unknown sample were added in a microplate, together with 225 μL of BCA working reagent. Then, the plate was incubated for 15 min at RT, and the absorbance was quantified at 750 nm in an automatic reader (Synergy 2, Agilent BioTek, Santa Clara, CA, USA).

### 4.11. Nanoparticle Track Analysis

Size distribution and particle concentration of purified vesicles were determined by Nanoparticle track analysis (NTA) in a NanoSight LM10-12 instrument (Malvern Instruments Ltd., Malvern, UK) equipped with a 638 nm laser and CCD camera (model F-033). Readings were taken in single capture for 60 s at 30 frames per second, at a camera level set to 680, and manual monitoring of temperature was conducted. Samples were diluted in PBS in order to obtain around 20 to 120 particles per frame. Data were analysed using the NTA software version 3.2.

### 4.12. Electron Microscopy

SEC fractions enriched in EVs were characterized by transmission electron microscopy (TEM) to further estimate their size and evaluate the morphology of isolated plasma EVs. For TEM, 7 μL of SEC pooled fractions were placed in a Ni-C 300 mesh grid and treated with uranyl acetate for 30 s. Images were acquired on a JEOL JEM 1400 transmission electron microscope (JEOL, Tokyo, Japan) and digitally recorded using a CCD digital camera Orius 1100W (Tokyo, Japan). Representative digital images of EVs from each sample were taken.

### 4.13. Samples Preparation and Liquid Chromatography Tandem Mass Spectrometry (LC-MS/MS)

After molecular characterization, the protein content of plasma-derived EVs was determined by mass spectrometry. These analyses were conducted by the Proteomics Scientific Platform at Instituto de Investigação e Inovação em Saúde (i3S). Briefly, samples (about 10 µg) were processed for proteomic analysis following the solid-phase-enhanced sample-preparation (SP3) protocol and enzymatically digested with trypsin/LysC as previously described [61]. Protein identification and quantitation were performed by nanoLC-MS/MS. 500 ng of peptides were injected in the Q-exactive equipment from ThermoFisher Scientific, Waltham, Massachusetts, EUA, using a separation system nano LC. This equipment is composed of an Ultimate 3000 liquid chromatography system coupled to a Q-Exactive Hybrid Quadrupole-Orbitrap mass spectrometer (Thermo Scientific, Bremen, Germany). Five hundred nanograms of peptides of each sample were loaded onto a trapping cartridge (Acclaim PepMap C18 100 Å, 5 mm × 300 μm i.d., 160454, Thermo Scientific, Bremen, Germany) in a mobile phase of 2% ACN, 0.1% FA at 10 μL/min. After 3 min loading, the trap column was switched in-line to a 50 cm × 75 μm inner diameter EASY- Spray column (ES903, PepMap RSLC, C18, 2 μm, Thermo Scientific, Bremen, Germany) at 250 nL/min. Separation was achieved by mixing A: 0.1% FA and B: 80% ACN, 0.1% FA with the following gradient: 5 min (2.5% B to 10% B), 120 min (10% B to 30% B), 20 min (30% B to 50% B), 5 min (50% B to 99% B), and 10 min (hold 99% B). Subsequently, the column was equilibrated with 2.5% B for 17 min. Data acquisition was controlled by Xcalibur 4.0 and Tune 2.11 software (Thermo Scientific, Bremen, Germany). The mass spectrometer was operated in the data-dependent (dd) positive acquisition mode alternating between a full scan (*m/z* 380–1580) and subsequent HCD MS/MS of the 10 most intense peaks from a full scan (normalized collision energy of 27%). The ESI spray voltage was 1.9 kV. The global settings were as follows: use lock masses best (*m/z* 445.12003), lock mass injection Full MS and chrom. peak width (FWHM) of 15 s. The full scan settings were as follows: 70 k resolution (*m/z* 200), AGC target 3 × 10^6^, maximum injection time 120 ms; dd settings: minimum AGC target 8 × 10^3^, intensity threshold 7.3 × 10^4^, charge exclusion: unassigned, 1, 8, >8, peptide match preferred, exclude isotopes on, and dynamic exclusion 45 s. The MS2 settings were as follows: microscans 1, resolution 35 k (*m/z* 200), AGC target 2 × 10^5^, maximum injection time 110 ms, isolation window 2.0 *m/z*, isolation offset 0.0 *m/z*, dynamic first mass, and spectrum data type profile. The raw data was processed using the Proteome Discoverer 2.5.0.400 software (Thermo Scientific) and searched against the UniProt database for the *Homo sapiens* Proteome (2021_03 with 20,371 entries), *Leishmania infantum* (2019_11 with 8045 entries) and HIV (2021_01) together with a spectral library database (NIST Human Orbitrap HCD 20160923). A common protein contaminant list from MaxQuant was also considered in the analysis. The MSPepSearch and Sequest HT search engines were used to identify tryptic peptides. The ion mass tolerance was 10 ppm for precursor ions and 0.02 Da for-fragment ions. The maximum allowed missing cleavage sites was set to two. Cysteine carbamidomethylation was defined as a constant modification. Methionine oxidation, deamidation of glutamine and asparagine, peptide terminus glutamine to pyroglutamate, and protein N-terminus acetylation, Met-loss, and Met-loss+acetyl were defined as variable modifications. Peptide confidence was set to high. The processing node Percolator was enabled with the following settings: maximum delta Cn 0.05; decoy database search target false discovery rate 1%, validation based on *q*-value. Protein label-free quantitation was performed with the Minora feature detector node at the processing step. Precursor ions quantification was performed at the consensus step with the following parameters: unique plus razor peptides were considered, precursor abundance was based on intensity, and normalization was based on total peptide amount. For hypothesis testing, protein ratio calculation was pairwise ratio-based and a *t*-test (background-based) hypothesis test was performed.

### 4.14. Quantitative Evaluation of Human Proteins

A quantitative analysis was also performed to compare the protein abundances in the three groups. From proteomic data, each protein has a numeric value for the abundance ratio between HIV^+^/HIV^−^VL^−^ controls; HIV^+^VL^+^/HIV^−^VL^−^ controls, and HIV^+^/HIV^+^VL^+^ and a *p*-value for each abundance value. This abundance ratio was calculated according to the number of unique peptides found for each protein in each group. Then, with this information were calculated which proteins are significantly over or underrepresented. A GO enrichment analysis using Database for Annotation, Visualization, and Integrated Discovery (DAVID 2021) and the proteins significantly over/underrepresented was performed [62].

### 4.15. Western Blot

EVs were denatured in 1× Laemmli buffer (0.25 M Tris-HCl, pH 6.8, 5% SDS, 20% glycerol 0.02% bromophenol blue, 2.5% β-Mercaptoethanol), for 10 min at 95 °C and separated on an 12% (*w*/*v*) acrylamide gel by SDS-PAGE following transfer onto a nitrocellulose/PVDF membrane using a Trans-Blot Turbo Transfer System (Bio-Rad, Hercules, CA, USA) ensued. The membrane was blocked with 5% (*w*/*v*) non-fat dried skimmed milk in PBS/0.1% Tween 20 for 1 h at RT. Then, the membrane was washed and incubated overnight with a 1:500 dilution of MARCO Antibody (AP9891A, Thermo Scientific) as primary antibody diluted in blocking solution overnight. Next, the membrane was washed and incubated with the secondary antibody diluted (1:10,000) in blocking solution (1010-05, SouthernBiotech, Birmingham, AL, USA) for 1h at RT. The membranes were then washed and rinsed in PBS and SuperSignal WestPico Chemiluminescent Substrate (Thermo Scientific) was added, protected from light, to cover the membrane surface. Amersham Hyperfilm ECL (Cytiva, Marlborough, MA, USA) Films were revealed from the membrane at specific times using the Fujifilm FPM-100A film processor (Fujifilm, Tokyo, Japan). All washes were performed at RT using PBS/0.1% Tween 20 (once for 15 min and 3 times for 5 min).

## Figures and Tables

**Table 1:** Clinical characterization of patient HIV^+^/VL^+^.

Sample	Collection Date	Sex	Age	Hospital	Clinical Aspects (Date Diagnostic)	Treatment	Parasitemia PCR (Copies/mL)
HIV^+^/VL^+^	16/05/19 (TP1)	M	45	Centro Hospitalar Universitário de São João, Porto, Portugal	HIV (2000) and VL (2013)	Lipossomal Amphotericin B and Miltefosine	5000
	14/01/20 (TP2)						6500
	30/07/20 (TP3)						7500
	14/01/21 (TP4)						3000
	29/07/21 (TP5)						7000

**Table 2:** *L. infantum* proteins identified in plasma-derived EVs from the five time points of the patient HIV^+^/VL^+^, either individually or after pooling. Depicted in the table are the identity of the sample, FDR, Uniprot accession number, description, number of peptides, peptide spectrum matches (PSMs), and unique peptides.

Sample	FDR	Accession (UniProtKB)	*Leishmania* Locus Tag	Description	PSMs	UPs
TP1	Medium	A4HSB7	LINJ_04_1200	Uncharacterized protein	1	1
	Medium	A4HUI7	LINJ_10_0800	Uncharacterized protein	1	1
TP2	Low	A4HTG6	LINJ_08_0160	GPI-GlcNAc transferase complex PIG-H component conserved domain-containing protein	1	1
TP3	Medium	A4I7M2	LINJ_32_0630	Uncharacterized protein	1	1
TP4	Medium	A4HVH9 **	LINJ_13_0390	Putative RNA helicase	1	1
TP5	Medium	A4HVL3	LINJ_13_0740	Uncharacterized protein	1	1
	Low	E9AHM5	LINJ_32_4140	Putative GIPL galf transferase	1	1
Pool HIV^+^VL^+^	High	A4HSC2 **	LINJ_04_1250	Actin	72	1
	High	A4I7Z7 *	LINJ_32_1910	Superoxide dismutase	3	1
	High	A4IA22 *	LINJ_34_2280	Uncharacterized protein	3	1
	High	A4I0Q2	LINJ_24_0650	Kinesin-like protein	1	1
	Medium	A4I2B2	LINJ_26_1790	HEAT repeat-containing protein 1	2	1
	Medium	A4I7L3	LINJ_32_0720	Small ribosomal subunit protein uS4 N-terminal domain-containing protein	4	1
	Medium	A4HW49	LINJ_14_1140	Protein kinase domain-containing protein	2	1
	Medium	A4HT30	LINJ_07_0090	RAVE complex protein Rav1 C-terminal domain-containing protein	4	1

* Found also in sample 3 of the validation cohort. ** Peptide not 100% specific for *Leishmania* upon BLAST analysis performed using NCBI blastp (version 2.16.0+/25 June 2024) for non-redundant protein sequences.

## Data Availability

The mass spectrometry proteomics data have been deposited to the ProteomeXchange Consortium via the PRIDE partner repository with the dataset identifier PXD049001.

## Supplementary Materials

The following supporting information can be downloaded at: https://www.mdpi.com/article/10.3390/ijms26125691/s1.
