# Supplementary material for: Proteomic Profiling Identifies MARCO in Extracellular Vesicles, as a Potential Biomarker for Leishmaniasis in HIV Co-Infection
Source: Int J Mol Sci. 2025 Jun 13;26(12):5691. doi: 10.3390/ijms26125691 (PMC12193235; doi:10.3390/ijms26125691)
Supplement: Supplementary file 1 [file ijms-26-05691-s001.zip › ijms-3615280-supplementary.pdf]

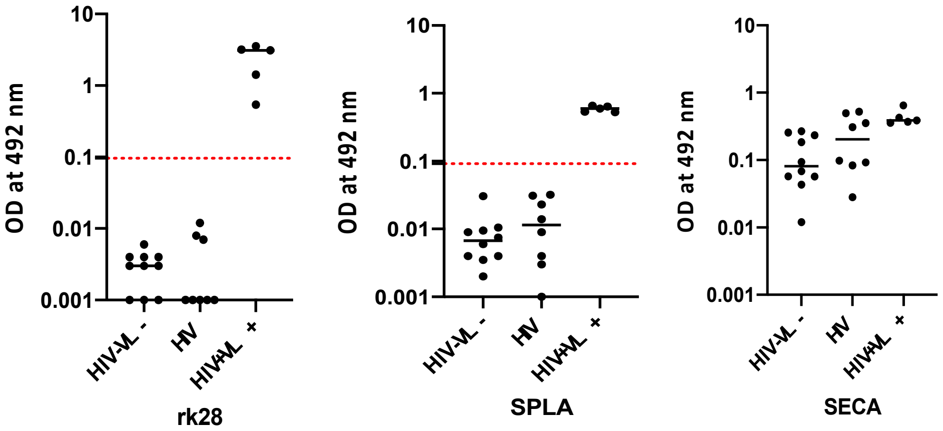

Figure S1. Seroreactivity from samples from three groups to SPLA, rK28 and SECA determined by ELISA. Results are represented as the optical density at 492 nm. Each dot represents a patient and the black solid line is the average of the ODs. The dashed red line represents the seropositivity cut-offs for the *Leishmania*-specific antigens, 0.096 for SPLA and 0.092 for rk28.

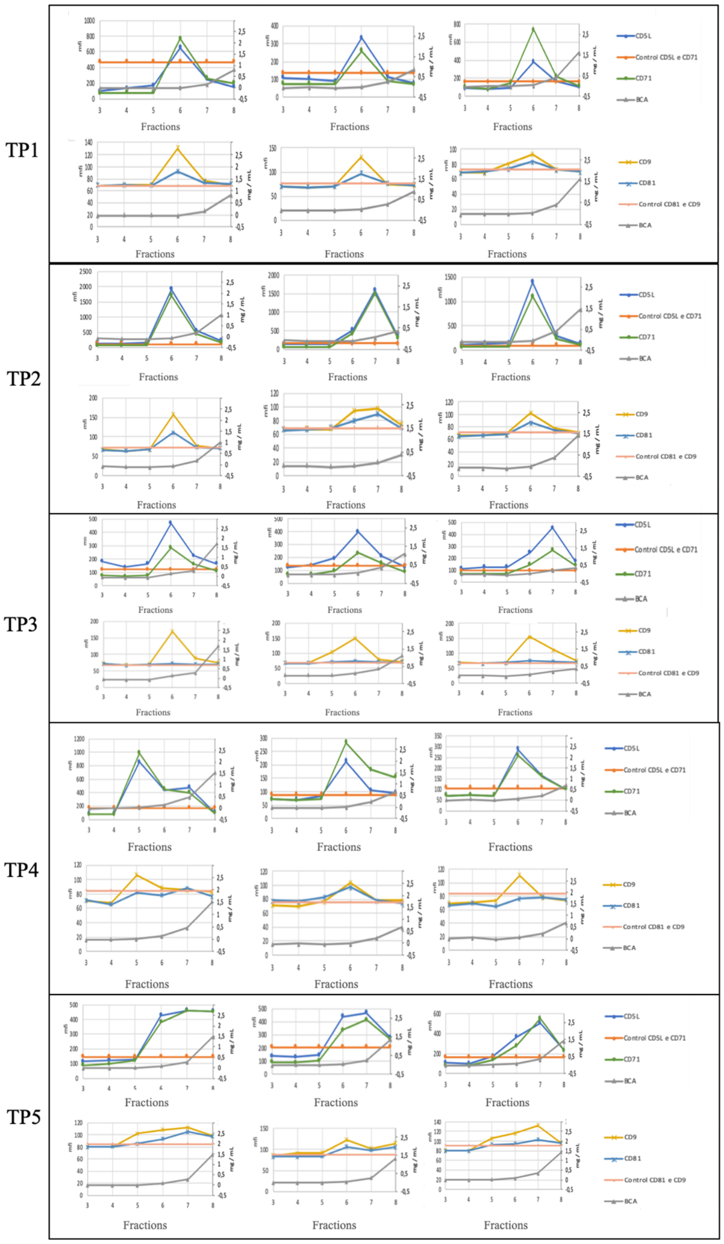

Figure S2. Fraction characterization using flow cytometry bead-based assay for the patient samples. SEC fractions 3 through 8 from the patient time points were analysed using a bead-based flow cytometry for the presence of EV-associated markers CD5L (light blue line); CD71 (green line) in the upper graphs and CD81 (dark blue line) and CD9 (yellow line) in the lower graphs. The presence of the antibodies is depicted by absolute MFI plotted on the left y-axis. In the right y-axis is plotted protein concentration for each fraction (BCA, grey line). As negative controls, pooled fractions (5 and 6) beads incubated with secondary antibodies only were used (orange line in the upper graph and light pink in the lower graphs).

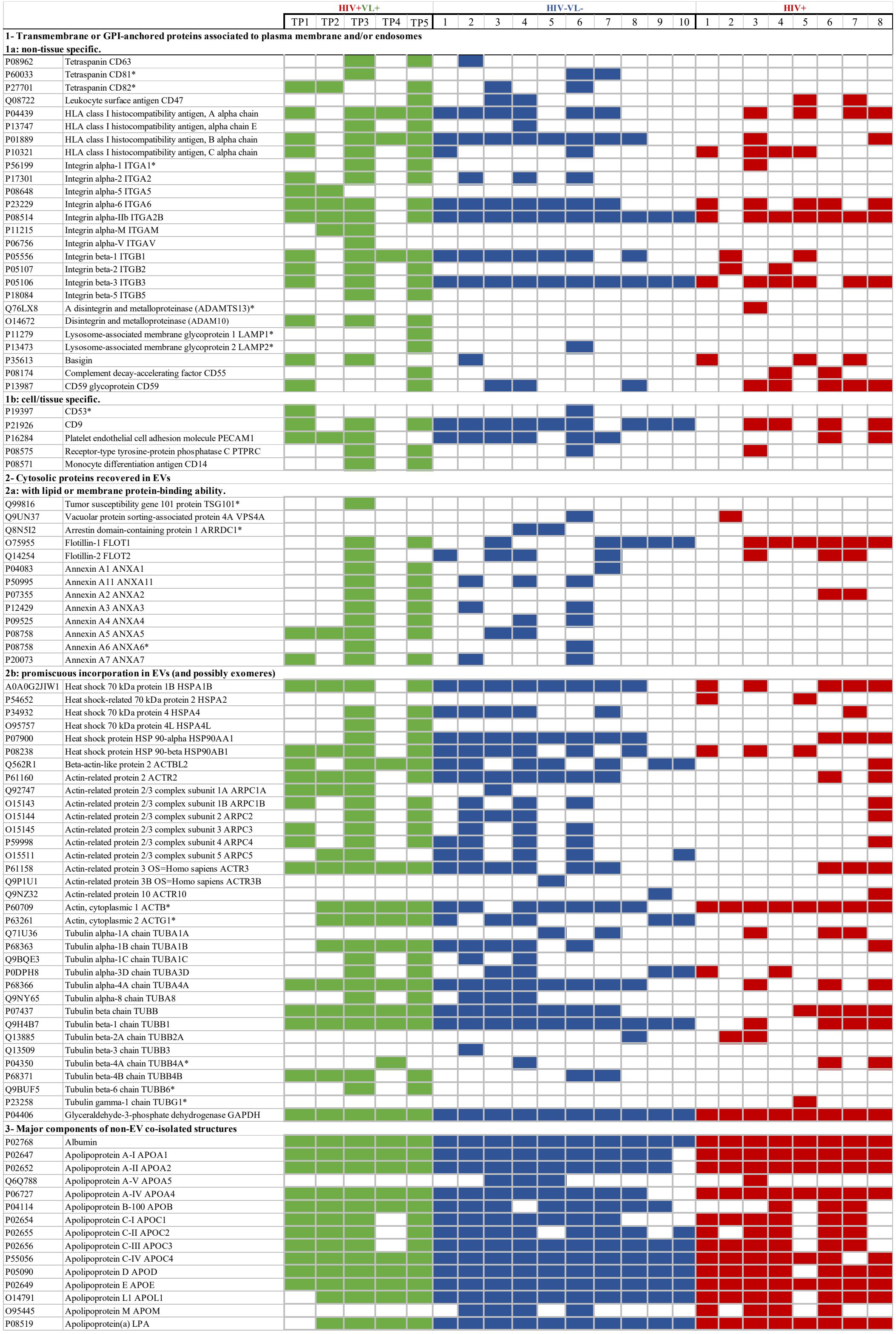

Figure S3. EV characterization based on the Misev2018 guidelines. Each line represents a protein, while the columns labelled with a number represent the individual samples. The presence of green (Time points from the HIV⁺VL⁺ patient), blue (HIV⁻VL⁻ control group), or red (HIV⁺ control group) colours associated with individual proteins depicted in lines represents the detection of that protein in the sample. * 1 Unique peptide.

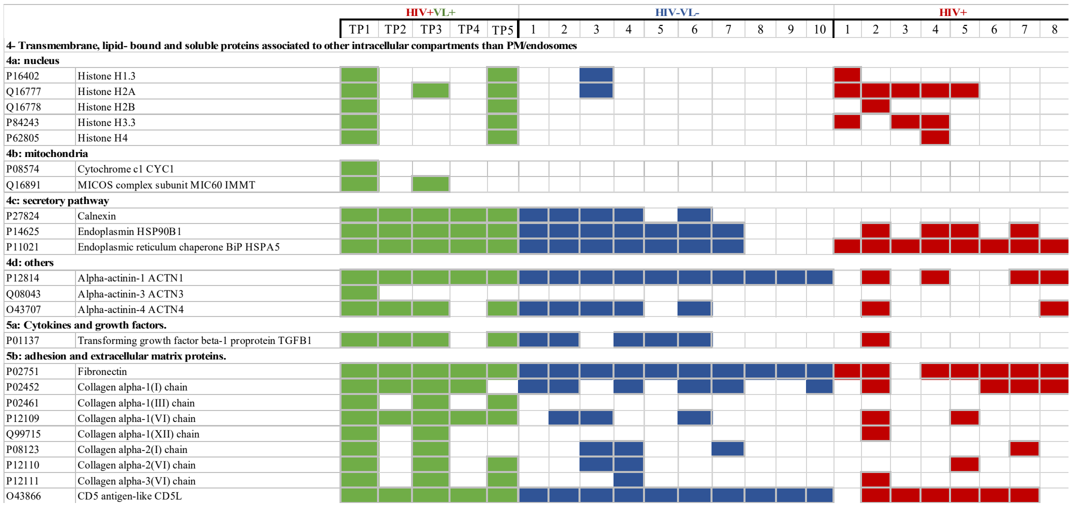

Figure S4. EVs characterization based on the category 4 and 5 of Misev2018 guidelines. Each line represents a protein, while the columns labelled with a number represent the individual samples. The presence of green (Time points from the $HIV^{+}VL^{+}$ patient), blue ($HIV^{-}VL^{-}$ control group), or red (HIV^{+} control group) colours associated with individual proteins depicted in lines represents the detection of that protein in the sample.

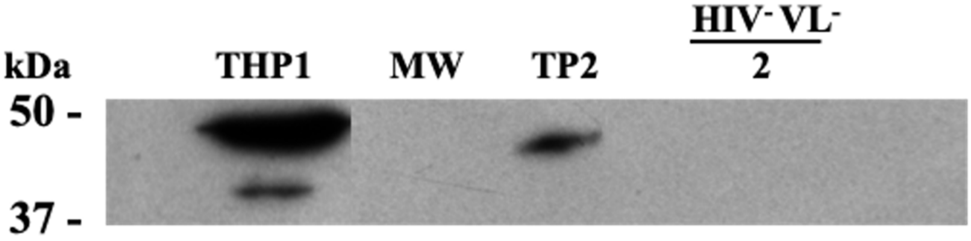

Figure S5. Detection of MARCO by western blot in plasma-derived EVs. MARCO antibody was used to detect MARCO in the plasma EVs from time point two of the patient HIV+VL+ (TP2) and $HIV^{-}VL^{-}$ control 2. For both samples, the EV fractions obtained from 500µl of plasma by SEC were concentrated to a final volume of 30µl using Vivaspin® 500 Centrifugal Concentrator (Sartorius, Germany), and the entire volume was loaded on the gel. THP-1 cell extract (60µg) was used as a positive control.

Table S1. Characteristics of Healthy ($HIV^{-}VL^{-}$) and HIV^{+} control groups.

	Healthy (HIV-VL-)										HIV (HIV+)							
**Sample**	1	2	3	4	5	6	7	8	9	10	1	2	3	4	5	6	7	8
**Age**	50	38	41	49	53	46	42	49	43	48	46	50	52	55	47	48	60	56
**Sex**	Male																	
**Nationality**	Portugal																	
**Hospital**	Centro Hospitalar Universitário São João, Porto, Portugal																	

Table S2. ROC curves for the Anti-*Leishmania* SPLA and rK28 with respective cut-offs, sensitivity (Sn), specificity (Sp), confidence interval (CI), and area under the curve (AUC).

	SPLA	RK28
**CUT-OFF**	0.092	0.096
**SN**	94.23	94.23
**SP**	94.23	98.08
**CI**	0.9165 to 1.003	0.9641 to 1.003
**AUC**	0.959	0.983

Table S3. Clinical characterization of validation cohort.

Sample	Collection Date	Sex	Age	Hospital	Status	Othe Diseases	Treatment	PCR Leishmania	rK39	Comments
Sample 1 (HIV+VL+)	20/12/2016	M	53	Hospital Universitario de Mósoles ,Madrid, Spain	IC	HIV	N/A	-	+	Spenomegaly
Sample 2 (HIV-VL+)	20/09/2019	M	2	Hospital Garcia de Ora, Almada, Portugal	N	N/A	Amphotericin B	+	N/A	Spenomegaly
Sample 3 (HIV-VL+)	23/01/2020	F	75	Centro Hospitalar Uniersitário São João, Porto, Portugal	IC	EBV; Herpes Simplex Type I	Amphotericin B	+	N/A	Pancytopenia; Bone Marrow Amastigotes
Sample 4 (HIV-VL+)	26/01/2017	M	80	Hospital Universitário Miguel Servet, Zaragoza, Spain	N/A	N/A	N/A	+	+	Previous VL, Cured, CL
Sample 5 (HIV+VL+)	01/02/2017	M	40	Hospital Virgen de la Victoria, Malaga, Spain	IC	N/A	N/A	+	+	Spenomegaly

N/A – Not available
IC – Immunocompromised
N - Immunocompetent

Table S4. *Leishmania* proteins found in other VL patients.

Sample	Protein FDR Confidence	Accession (UniProtKB)	Leishmania Locus Tag	Descritpion	#PSMs	#UPs
Sample 1 (HIV+VL+)	Medium	A4IBW7	LINJ_35_4360	Conserved hypothetical protein	1	1
	High	E9AHS2	LINJ_34_4110	Ankyrin repeats (3 copies), putative	1	1
	High	A4I5T0	LINJ_30_2540	Putative heat shock 70-related protein 1, mitochondrial	1	1
	High	A4I2W5	LINJ_27_1650	Putative dynein heavy chain	1	1
	High	A4I198	LINJ_25_0580	Protein-tyrosine-phosphatase, putative	1	1
	Medium	Q9GNZ8	LINJ_21_2070	Proteasome subunit alpha type	1	1
	High	A4HWQ0	LINJ_16_0020	Large ribosomal subunit protein uL15/eL18 domain-containing protein	1	1
Sample 2 (HIV-VL+)	High	A4HUK3	LINJ_10_0960	Putative small GTP-binding protein Rab11	2	1
	Medium	A4I0K2	LINJ_23_1700	Palmitoyltransferase	1	1
Sample 3 (HIV-VL+)	High	A4I7Z7	LINJ_32_1910	Superoxide dismutase	2	1
	Medium	A4IA22	LINJ_34_2280	Uncharacterized protein	3	1

Table S5. List of patient proteins with significantly increased abundance. The table depicts the Uniprot accession number of proteins that were detected with increased abundance in the patient (HIV$^+$VL$^+$) in comparison to both control groups.

Accession (UniProtKB)	Description
Q13751	Laminin subunit beta-3
Q9H1H9	Kinesin-like protein KIF13A
Q9H3P7	Golgi resident protein GCP60
P06241	Tyrosine-protein kinase Fyn
Q05655	Protein kinase C delta type
O95182	NADH dehydrogenase [ubiquinone] 1 alpha subcomplex subunit 7
Q9Y4F1	FERM, ARHGEF and pleckstrin domain-containing protein 1
P84085	ADP-ribosylation factor 5
P17252	Protein kinase C alpha type
P84085	ADP-ribosylation factor 5
Q4V328	GRIP1-associated protein 1
P78386	Keratin, type II cuticular Hb5
O15231	Zinc finger protein 185
Q86UP2	Kinectin
P51692	Signal transducer and activator of transcription 5B
P55735	Protein SEC13 homolog
Q92747	Actin-related protein 2/3 complex subunit 1A Q9Y262
Q9Y262	Eukaryotic translation initiation factor 3 subunit L
O95671	Probable bifunctional dTTP/UTP pyrophosphatase/methyltransferase protein
P49767	Vascular endothelial growth factor C
Q96IZ0	PRKC apoptosis WT1 regulator protein
Q15041	ADP-ribosylation factor-like protein 6-interacting protein 1 P98172
P98172	Ephrin-B1
P51809	Vesicle-associated membrane protein 7
Q6EEV6	Small ubiquitin-related modifier 4

O00116	Alkyldihydroxyacetonephosphate synthase, peroxisomal Q15389
Q15389	Angiopoietin-1
Q9UBW8	COP9 signalosome complex subunit 7a
B2RUZ4	Small integral membrane protein 1
Q9NRL3	Striatin-4
Q5VY43	Platelet endothelial aggregation receptor 1
Q9HD45	Transmembrane 9 superfamily member 3
Q96A33	PAT complex subunit CCDC47
P41227	N-alpha-acetyltransferase 10
P46459	Vesicle-fusing ATPase
Q9NZQ3	NCK-interacting protein with SH3 domain
Q9Y376	Calcium-binding protein 39
Q96QR8	Transcriptional activator protein Pur-beta
Q16822	Phosphoenolpyruvate carboxykinase [GTP], mitochondrial
Q96HS1	Serine/threonine-protein phosphatase PGAM5, mitochondrial
Q8WUA8	Tsukushi 3
O94929	Actin-binding LIM protein 3
P14735	Insulin-degrading enzyme
Q9NZ43	Vesicle transport protein USE1
Q9NZZ3	Charged multivesicular body protein 5
P53801	Pituitary tumor-transforming gene 1 protein-interacting protein
P09417	Dihydropteridine reductase
P16333	Cytoplasmic protein NCK1
Q9HAT2	Sialate O-acetylesterase
P30566	Adenylosuccinate lyase
Q9BY44	Eukaryotic translation initiation factor 2A
Q8WU79	Stromal membrane-associated protein 2
Q96P50	Arf-GAP with coiled-coil, ANK repeat and PH domain-containing protein 3 Q9UKK9
Q9UKK9	ADP-sugar pyrophosphatase
Q8NG11	Tetraspanin-14
Q08379	Golgin subfamily A member 2
Q96AG4	Leucine-rich repeat-containing protein 59
Q9Y5I0	Protocadherin alpha-13
P29279	CCN family member 2
Q9Y2X7	ARF GTPase-activating protein GIT1
P15586	N-acetylglucosamine-6-sulfatase
Q9BUP3	Oxidoreductase HTATIP2
O00330	Pyruvate dehydrogenase protein X component, mitochondrial
Q6ZSS7	Major facilitator superfamily domain-containing protein 6 P78559
P78559	Microtubule-associated protein 1A
Q5VZK9	F-actin-uncapping protein LRRC16A
Q9UI42	Carboxypeptidase A4
Q02040	A-kinase anchor protein 17A
Q4G0F5	Vacuolar protein sorting-associated protein 26B O95059
O95059	Ribonuclease P protein subunit p14
Q2WGJ9	Fer-1-like protein 6

.

Table S6. List of patient proteins with significantly decreased abundance. The table depicts the Uniprot accession number of proteins that were detected with increased abundance in the patient (HIV$^+$VL$^+$) in comparison to both control groups.

Accession (UniProtKB)	Description
Q7Z7G0	Protein phosphatase 1H
P49643	Solute carrier family 2, facilitated glucose transporter member 2
P00740	Cullin-1
Q9NXC5	[Protein ADP-ribosylarginine] hydrolase-like protein 1
P35030	Trypsin-3
Q8WWQ8	Mitochondrial intermediate peptidase
Q6UWD8	Voltage-dependent calcium channel subunit alpha-2/delta-2
Q15434	Cathepsin Z
P07148	Poly(U)-specific endoribonuclease
Q9H223	Protein broad-minded
Q9ULQ0	Complex III assembly factor LYRM7
Q9UHQ4	5-oxoprolinase
Q06278	U2 snRNP-associated SURP motif-containing protein
Q5VYK3	Sideroflexin-4
O14744	Argininosuccinate lyase
O43617	Semenogelin-2
Q6KB66	Epoxide hydrolase 1
P08572	Immunoglobulin lambda variable 4-3
A0FGR8	Keratin, type I cytoskeletal 23
Q96DR8	E3 ubiquitin-protein ligase TRIM9
Q9Y5K6	Zinc finger protein castor homolog 1 Q86VP6
Q86VP6	Fructose-bisphosphate aldolase B
P07602	FHF complex subunit HOOK interacting protein 1B
Q9UJM8	Endoplasmic reticulum mannosyl-oligosaccharide 1,2-alpha-mannosidase
P10253	Mucin-2
Q9UNZ2	Polypeptide N-acetylgalactosaminyltransferase 3
P14317	General transcription factor IIF subunit 2
P13984	Hematopoietic lineage cell-specific protein
Q14435	NSFL1 cofactor p47
Q02817	Lysosomal alpha-glucosidase
Q9UKM7	Hydroxyacid oxidase 1
Q8N612	Prosaposin
P05062	Cullin-associated NEDD8-dissociated protein 1
Q86V15	CD2-associated protein
Q9C026	Mucin-like protein 1
Q9C075	Extended synaptotagmin-2
A0A075B6K6	Collagen alpha-2(IV) chain
P07099	Keratin, type II cytoskeletal 80
Q02383	Trafficking protein particle complex subunit 3 P04424
P04424	Protein arginine N-methyltransferase 5
Q6P4A7	Proteasome adapter and scaffold protein ECM29
P15088	Mast cell carboxypeptidase A
O15042	Aldehyde oxidase
O14841	B-cell receptor-associated protein 29
Q5U5X0	Striatin-interacting protein 2
Q96NH3	EH domain-containing protein 4

P21128	Fatty acid-binding protein, liver
Q9UBR2	RNA-binding motif, single-stranded-interacting protein 2
Q9NY47	Transmembrane protein C16orf54
Q99797	Stabilin-2
Q9GZU2	Paternally-expressed gene 3 protein Q8NDY3
Q8NDY3	GATOR complex protein MIOS Q13616
Q13616	Coagulation factor IX
P11168	DNA primase large subunit
Q9ULR3	Target of Nesh-SH3
Q15208	Serine/threonine-protein kinase 38
O00422	Histone deacetylase complex subunit SAP18
Q6IE36	Ovostatin homolog 2
P51884	Lumican
A0A0C4DH29	Immunoglobulin heavy variable 1-3 Q5T6V5
Q5T6V5	Queuosine salvage protein
O14936	Peripheral plasma membrane protein CASK

Table S7. Biological processes identified by GO enrichment analysis, using the Database for Annotation, Visualization, and Integrated Discovery (David 2021), associated with proteins with significantly increased or decreased abundance for each comparison considering the numerator. Only GO terms with p-value <0,05 are considered.

	GO Terms (Biological Process)	Count	Pvalue	Fold Enrichment
	**Significantly overrepresented in ratio HIV+VL+/HIV+**			
GO: 0009060	aerobic respiration	6	0.0012	7.5567
GO: 0006120	mitochondrial electron transport, NADH to ubiquinone	5	0.0019	9.3913
GO: 0051603	proteolysis involved in protein catabolic process	5	0.0022	9.008
GO: 0050790	regulation of catalytic activity	12	0.038	2.8099
GO: 0006886	intracellular protein transport	11	0.0043	2.9516
GO: 0023035	CD40 signaling pathway	3	0.0054	26.4836
GO: 0002474	antigen processing and presentation of peptide antigen via MHC class I	3	0.0054	26.4836
GO: 0046513	ceramide biosynthetic process	4	0.006	10.7004
GO: 0042776	proton motive force-driven mitochondrial ATP synthesis	5	0.0062	6.7907
GO: 0019885	antigen processing and presentation of endogenous peptide antigen via MHC class I	3	0.0065	24.076
GO: 0042113	B cell activation	4	0.0077	9.8087
GO: 0097242	amyloid-beta clearance	3	0.0091	20.372
GO: 0043248	proteasome assembly	3	0.0091	20.372
GO: 0073001	cellular response to hydrogen peroxide	5	0.0098	5.9648
GO: 0008631	intrinsic apoptotic signaling pathway in response to oxidative stress	3	0.0154	15.5786
GO: 0070374	positive regulation of ERK1 and ERK2 cascade	8	0.0154	3.084
GO: 0043066	negative regulation of apoptotic process	13	0.0179	2.1653
GO: 0043536	positive regulation of blood vessel endothelial cell migration	4	0.0188	7.0623
GO: 0008333	endosome to lysosome transport	4	0.0188	7.0623
GO: 0006508	proteolysis	11	0.0206	2.3231
GO: 0032874	ositive regulation of stress-activated MAPK cascade	3	0.021	12.2418
GO: 0042542	response to hydrogen peroxide	4	0.0219	6.6625
GO: 0071222	cellular response to lipopolysaccharide	7	0.0224	3.2018
GO: 0042340	keratan sulfate catabolic process	2	0.0224	88.2785

GO:	0071356	cellular response to tumor necrosis factor	6	0.025	3.6279
GO:	0010629	negative regulation of gene expression	9	0.0274	2.5143
GO:	0045807	positive regulation of endocytosis	3	0.0274	11.5146
GO:	0014823	response to activity	4	0.0277	6.0882
GO:	0046718	symbiont entry into host cell	5	0.0284	4.3274
GO:	0051289	protein homotetramerization	4	0.029	5.985
GO:	0022900	electron transport chain	4	0.029	5.985
GO:	0045861	negative regulation of proteolysis	3	0.0297	11.0348
GO:	0030335	positive regulation of cell migration	8	0.0305	2.6751
GO:	1900148	negative regulation of Schwann cell migration	2	0.0334	88.8524
GO:	0001934	positive regulation of protein phosphorylation	7	0.0335	2.9149
GO:	0032981	mitochondrial respiratory chain complex I assembly	4	0.0343	5.605
GO:	0043524	negative regulation of neuron apoptotic process	6	0.0344	3.3313
GO:	0046686	response to cadmium ion	3	0.0395	9.4584
GO:	0006911	phagocytosis, engulfment	5	0.0413	3.8382
GO:	0010595	positive regulation of endothelial cell migration	4	0.0415	5.1929
GO:	0006853	carnitine shuttle	2	0.0444	44.1393
GO:	0010992	ubiquitin recycling	2	0.0444	44.1393
GO:	0036018	cellular response to erythropoietin	2	0.0444	44.1393
GO:	0030163	protein catabolic process	4	0.0446	5.0445
GO:	0043162	ubiquitin-dependent protein catabolic process via the multivesicular body sorting pathway	3	0.0475	8.5431
		**Significantly overrepresented in ratio HIV+VL+/HIV-**			
GO:	0015031	protein transport	11	0.017	3.3409
GO:	0006888	endoplasmic reticulum to Golgi vesicle-mediated transport	6	0.003	6.1068
GO:	0006413	translational initiation	4	0.072	10.062
GO:	0045109	intermediate filament organization	4	0.0132	8.0807
GO:	0000226	microtubule cytoskeleton organization	5	0.0189	4.9019
GO:	0060548	obsolete negative regulation of cell death	4	0.0205	6.8375
GO:	0032147	activation of protein kinase activity	4	0.0219	6.6666
GO:	0035987	endodermal cell differentiation	3	0.0222	12.903
GO:	0090314	positive regulation of protein targeting to membrane	3	0.0222	12.903
GO:	0007010	cytoskeleton organization	5	0.0238	4.5661
GO:	0008544	epidermis development	4	0.0257	6.2744
GO:	0030032	lamellipodium assembly	3	0.0279	11.428
GO:	1903912	negative regulation of endoplasmic reticulum stress-induced eIF2 alpha phosphorylation	2	0.0367	53.3324
GO:	0032728	positive regulation of interferon-beta production	3	0.0391	9.52364
		**Significantly overrepresented in ratio HIV+VL+/HIV- and HIV+VL+/HIV+**			
GO:	0030032	lamellipodium assembly	3	0.0279	11.4284
GO:	0043001	Golgi to plasma membrane protein transport	3	0.0196	13.7929
GO:	0048013	ephrin receptor signaling pathway	4	0.0065	10.4573
GO:	0042447	hormone catabolic process	2	0.0295	66.6655
		**Significantly underrepresented in ratio HIV+VL+/HIV+**			
GO:	0006897	endocytosis	6	0.0093	4.6473
		**Significantly underrepresented in ratio HIV+VL+/HIV-VL-**			
GO:	0005975	carbohydrate metabolic process	5	0.0218	4.6833
GO:	0006979	response to oxidative stress	4	0.0387	5.3258
GO:	0051260	protein homooligomerization	4	0.0437	5.0742
GO:	0061684	chaperone-mediated autophagy	2	0.0482	40.277

Table S8. Peptide sequence from Marco protein found in preparations of plasma Evs from dogs with Canine Leishmaniosis.

Marco peptide identified in four Leishmania infected dogs in Esteves et al [15]
[K].SSWGSHNCNHSEDAGVECS.[-]
